# Diagnostic value of tongue manifestations for primary Sjögren’s syndrome: construction and validation of disease screening model

**DOI:** 10.3389/fmed.2025.1552781

**Published:** 2025-05-27

**Authors:** Jia-qi Chen, Yan Zhang, Jia-he Liao, Jian-ying Yang, Bo-han Jiang, Zi-wei Huang, Tzu-hua Wu, Li-ning Zhang, Zi-han Wang, Chun-xin Lei, Xi-ya Zhang, Jing Luo, Qing-wen Tao

**Affiliations:** ^1^Graduate School, Beijing University of Chinese Medicine, Beijing, China; ^2^Traditional Chinese Medicine Department of Rheumatism, China-Japan Friendship Hospital, Beijing, China; ^3^Beijing Key Laboratory of Immune Inflammatory Disease, Beijing, China

**Keywords:** Sjögren’s syndrome, tongue, traditional Chinese medicine, screening model, diagnosis

## Abstract

**Objective:**

Tongue manifestations (tongue fur and tongue body) of traditional Chinese medicine is specific for primary Sjögren’s syndrome (pSS). We investigated the value of tongue manifestations in the diagnosis of pSS and established a model for screening or diagnosing pSS.

**Methods:**

A total of 400 individuals attended at the China-Japan Friendship Hospital were included in this study. 200 patients with pSS and 200 healthy individuals were randomly divided into training and test sets (7:3 ratio). The training set was used to build models based on combined tongue, tongue fur, and tongue body, respectively, and the optimal model was selected through model discrimination and calibration. The optimal model is internally validated using the bootstrap method and further performed on the test set to assess transportability and generalisability. Receiver operating characteristic curve, calibration curve, Brier score, and decision curve analysis (DCA) were used to evaluate the performance of the model.

**Results:**

In the training set, we identified an optimal model which included sex, age, bluish-purple tongue, thin tongue, fissured tongue, thin fur, and peeling fur, after evaluating and comparing three models. The model demonstrated an area under the curve (AUC) of 0.89, and the lowest Brier score of 0.13, with well-fitted calibration curves. The optimal cut-off value for the model is 0.347, with sensitivity and specificity of 87.86 and 76.43%, respectively. The model also shows well discrimination in internal validation. In the test set, this model demonstrated an AUC of 0.93 and with a Brier score of 0.11, and sensitivity and specificity of 93.33 and 71.67%, respectively. A nomogram for pSS screening was developed based on the model.

**Conclusion:**

The non-invasive, reliable and convenient model, established based on age and sex of the patient and specific tongue manifestations can serve as a clinical reference tool for screening or diagnosing of pSS.

## 1 Introduction

Primary Sjögren’s syndrome (pSS) is a systemic autoimmune disease, characterized by lymphocyte infiltration, that primarily affects the salivary and lacrimal glands (1). pSS also presents diverse extraglandular manifestations, contributing to the variability in disease classification (2). Since the first diagnostic classification criteria for Sjögren’s syndrome (SS) were established in 1965, there have been 13 different sets of criteria proposed (3–15). Among these, the American-European Consensus Group Classification criteria (AECG) are widely used and include two subjective symptoms among the six assessment items (12). The 2012 American College of Rheumatology (ACR) SS classification removed the influence of subjective symptoms and improves the specificity of diagnosis (13). 2016 American College of Rheumatology/European League against Rheumatism Classification Criteria (ACR-EULAR) placed more emphasis on extraglandular manifestations compared to previous classifications (15). Despite these updates, minor salivary gland biopsy (MSGB) and serum autoantibody testing remain critical for pSS diagnosis across these criteria. For fear of biopsy or lack of antibody detection and pathological diagnostic conditions, some potential pSS patients cannot be diagnosed (16).

In pSS patients, tongue manifestations are commonly observed and specific to the disease (17–21). Changes in the structure and color of the tongue can be important indicators for the diagnosis of SS (20). Reduced salivary gland function leads to significant decreases in oral saliva production and changes in saliva composition, resulting in symptoms such as dry mouth, atrophy of tongue papillae, dry lips, and absence of saliva accumulation under the tongue (22). In traditional Chinese medicine (TCM), the tongue is an important diagnostic tool, which can reflect the body’s physiological and clinicopathological condition (23). Due to its simplicity and accessibility, tongue diagnosis is widely used in TCM (24, 25). pSS patients often exhibit distinctive tongue features, predominantly fissured and dark purple tongues (17). These specific manifestations of tongue provide a non-invasive, simple, and effective potential screening or diagnostic tool for the disease.

Currently, research on the use of tongue diagnosis for pSS screening is limited. A small-scale study has indicated that diagnostic sensitivities and specificities of over 70% can be achieved using machine learning methods for non-contact tongue diagnosis in SS (18). However, this study included only 60 patients and excluded male participants. Considering the promising potential and clinical need for tongue manifestations in the screening and diagnosis of pSS, this study compared the effectiveness of several models based on tongue manifestations, selected and validated the optimal model, and evaluated its clinical applicability, so as to provide clinicians with a reliable and convenient screening or diagnosis method for pSS patients.

## 2 Materials and methods

### 2.1 Study design

This study was approved by the Clinical Research Ethics Committee of the China-Japan Friendship Hospital as part of a cross-sectional study (2021-144-K102).

### 2.2 Sample size calculation

**Figure 1:**
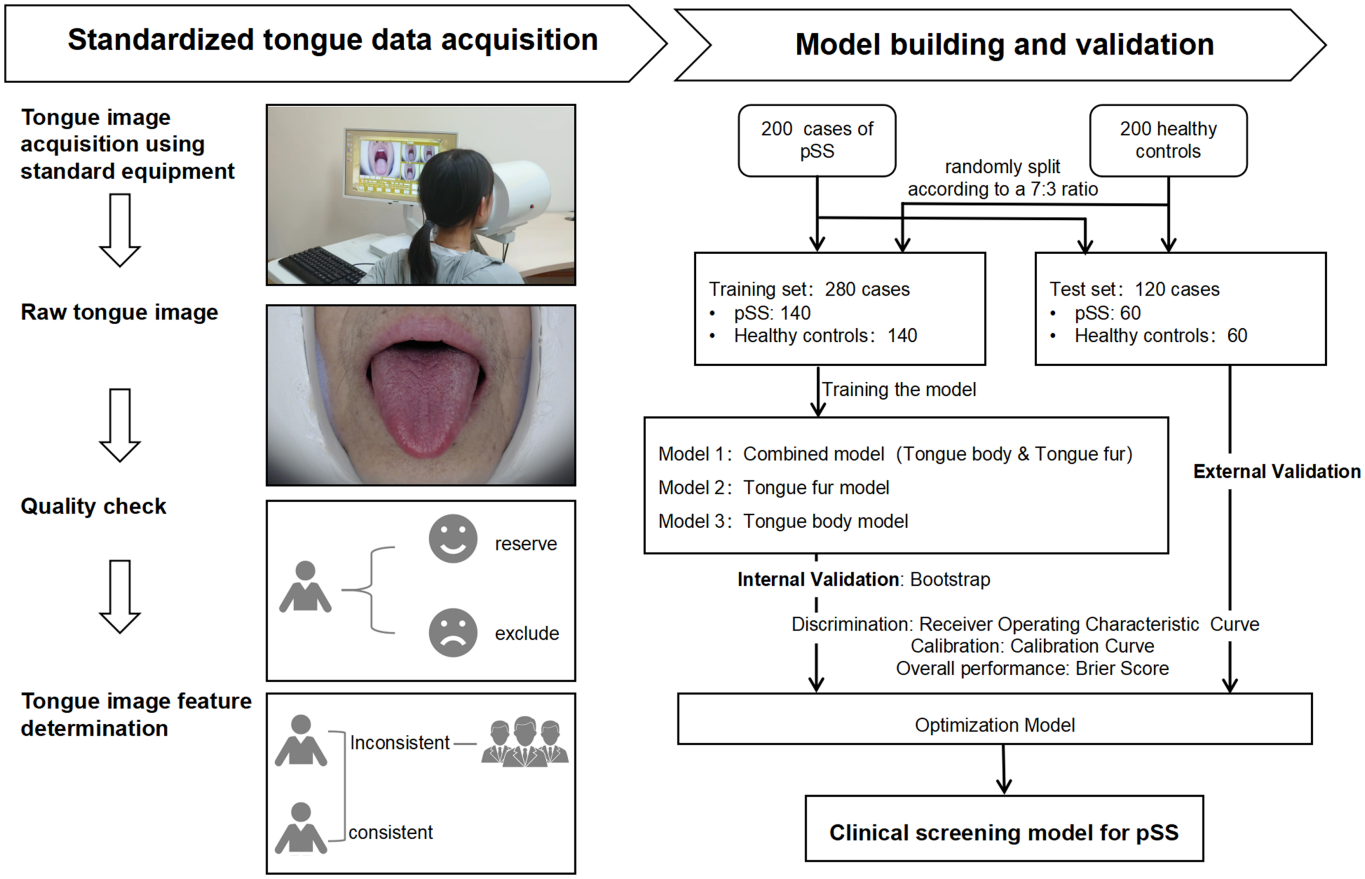
Flowchart for construction of the diagnostic model for primary Sjögren’s Syndrome. pSS: Primary Sjögren’s Syndrome.

A total of 17 candidate variables were included in this study. Assuming that 10–14 candidate variables are related to the diagnosis of pSS, and based on the rule of 10 events per variable in clinical prediction model (26), the sample size of patients with a diagnosis of pSS in this study was 100–140 cases. The ratio of pSS patients to healthy individuals was 1:1, requiring 200–280 participants in total. The actual number of participants included was 400, with 200 cases in each group. The subjects were split according to a 7:3 ratio, with 280 cases in the training set and 120 cases in the test set. See Figure 1 for the construction of the model.

### 2.3 Patients

Patients with pSS attending the TCM Department of Rheumatism and healthy individuals attending the physical examination center at the China-Japan Friendship Hospital (a tertiary care hospital) from December 2021 to December 2023 were included in this study. The inclusion criteria for this study were as follows: (1) pSS patients meet the 2016 ACR-EULAR classification criteria (15) for pSS; (2) age 18–75 years and (3) consent to taking tongue images. The exclusion criteria of this study were as follows: (1) comorbidity with other connective tissue diseases such as rheumatoid arthritis and systemic lupus erythematosus; (2) comorbidity with tumors, infection, serious cardiovascular or cerebrovascular diseases, and metabolic diseases; (3) long-term use of antibiotics or hormones, (4) oral diseases such as gum diseases.

### 2.4 Data collection

**Figure 2:**
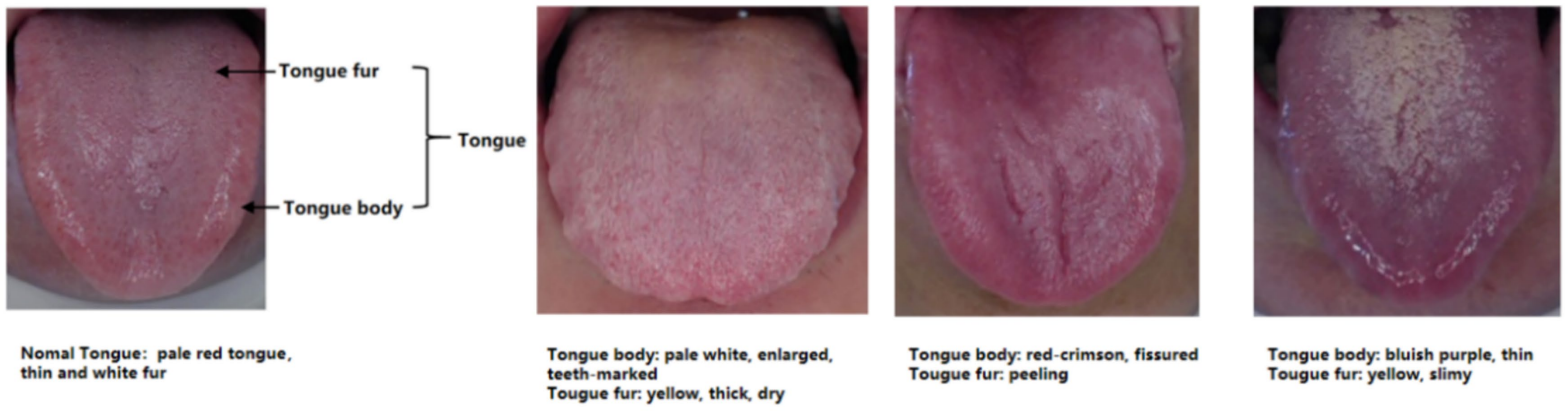
Schematic of tongue appearance.

Age, sex at birth, and tongue images were collected from participants in both groups. When the tongue images of the participants were being taken, the participants were required to sit in an upright position with their mouths as wide open as possible and their tongues naturally extended. The researcher used the DS01-B Tongue Information Acquisition System to take images of the tongue. On the day the tongue images were taken, the researcher confirmed with the participant that he/she had not consumed any food or medication that could change the color of the tongue body or tongue fur, and that he/she had not eaten or drunk any food or water 1 h before the image was taken. At the same time, participants were instructed to disclose all pharmacologic agents in use. A researcher checked the integrity of the tongue pictures taken. Tongue characteristics were determined by two senior physicians with extensive experience in the clinical diagnosis and treatment of TCM rheumatism. In the situation of disagreement between the two physicians, the decision was made after a team discussion. Two dimensions of the tongue image were recorded, namely, tongue body and tongue fur. Tongue body manifestations included pale red tongue, pale white tongue, red-crimson tongue, bluish-purple tongue, enlarged tongue, thin tongue, fissured tongue, and teeth-marked tongue; tongue fur manifestations included white fur, yellow fur, thick fur, thin fur, slimy fur, dry fur, and peeling fur. Among the tongue body manifestations, the red-crimson tongue manifestation included red tongue or crimson tongue. The bluish-purple tongue manifestation involved a color change to blue or purple across the entire tongue, and also included parts of the tongue that appeared to show bluish-purple spots, of different sizes, under the surface of the tongue. No tongue image data was missing in this study. The acquisition of standardized data is shown in Figure 1, and the diagram of tongue is shown in Figure 2.

### 2.5 Establishment and validation of the clinical diagnostic model

First, the tongue factors were analyzed by one-way analysis. Tongue factors with *p* values less than 0.1 in the one-way analysis were included for covariance analysis and binary logistic regression. Three models were developed for tongue characteristics; among these, model 1 included tongue body and tongue fur; model 2 included tongue fur only; and model 3 included only the tongue body. All three models included gender and age as correction factors. All models were further screened and optimized using the Akaike information criterion (AIC) combined with stepwise regression. Model discrimination was assessed by receiver operating characteristic (ROC) curves, model calibration was assessed through calibration plots, overall performance of the model was evaluated by Brier score, and the clinical applicability of the model was evaluated by decision curve analysis (DCA). Internal validation was performed by the bootstrap method. The optimal model among the three models was selected based on differentiation and calibration, and further validated in the test set. The optimal model used the training set data to calculate the Jordan index to find the optimal cutoff value. The sensitivity, specificity, positive predictive value, and negative predictive value of the optimal model were calculated in both the training and test sets.

### 2.6 Model presentation

Forest and nomogram of the optimal model were drawn to make the model easier to interpret.

### 2.7 Statistical methods

SPSS 27.0 and R4.3.2 software packages were used for statistical analysis. Count data were presented as frequency and percentage, while non-normally distributed data were represented by median and quartile (Q1, Q3). In group comparisons, the chi-square test was used for count data, and the Mann–Whitney U test was used for measures that did not conform to normal distribution. R4.3.2 was used for randomized splitting of datasets, model construction, model validation, model testing, and model presentation. Hypothesis testing was performed using two-sided test, and *p* < 0.05 was considered to indicate statistical significance.

## 3 Results

### 3.1 Tongue analysis

The results of one-way analysis of the data for the two groups among the overall participants and the training set are shown in Table 1. In the training set, the results indicate differences in terms of sex, age, pale red tongue, bluish-purple tongue, thin tongue, fissured tongue, teeth-marked tongue, thin fur, thick fur, dry fur, slimy fur, peeling fur, and white fur (*p* < 0.1). These factors were incorporated into the development of subsequent diagnostic models.

**Table 1:** Basic clinical information for the total study population.

Characteristic	Total				Training set		
	Total *N* = 400	pSS *N* = 200	Healthy controls *N* = 200	*P* value	set pSS *N* = 140	Healthy controls *N* = 140	*P* value
Sex at birth [*n* (%)]							
Male	48 (12)	14 (7.0)	34 (17)	0.002	11 (7.9)	22 (16)	0.041
Female	352 (88)	186 (93)	166 (83)		129 (92)	118 (84)	
Age [Median (IQR)]	50 (40, 59)	57 (50, 63)	42 (35, 51)	<0.001	57 (48, 63)	42 (34, 51)	<0.001
Tongue body [*n* (%)]							
Pale red tongue	133 (33)	51 (26)	82 (41)	0.001	33 (24)	57 (41)	0.002
Pale tongue	15 (3.8)	5 (2.5)	10 (5.0)	0.188	4 (2.9)	8 (5.7)	0.238
Red-crimson tongue	122 (31)	66 (33)	56 (28)	0.277	45 (32)	41 (29)	0.604
Bluish-purple tongue	168 (42)	98 (49)	70 (35)	0.005	71 (51)	47 (34)	0.004
Enlarged tongue	90 (23)	50 (25)	40 (20)	0.231	35 (25)	32 (23)	0.674
Thin tongue	43 (11)	37 (19)	6 (3.0)	<0.001	23 (16)	5 (3.6)	<0.001
Fissured tongue	150 (38)	118 (59)	32 (16)	<0.001	82 (59)	22 (16)	<0.001
Teeth-marked tongue	101 (25)	43 (22)	58 (29)	0.084	30 (21)	47 (34)	0.023
Tongue fur [*n* (%)]							
Thin fur	236 (59)	73 (37)	163 (82)	<0.001	54 (39)	118 (84)	<0.001
Thick fur	134 (34)	97 (49)	37 (19)	<0.001	64 (46)	22 (16)	<0.001
Dry fur	82 (21)	58 (29)	24 (12)	<0.001	42 (30)	14 (10)	<0.001
Slimy fur	152 (38)	99 (50)	53 (27)	<0.001	66 (47)	37 (26)	<0.001
Peeling fur	89 (22)	84 (42)	5 (2.5)	<0.001	61 (44)	4 (2.9)	<0.001
White fur	258 (65)	104 (52)	154 (77)	<0.001	74 (53)	107 (76)	<0.001
Yellow fur	114 (29)	68 (34)	46 (23)	0.015	45 (32)	33 (24)	0.110

pSS: Primary Sjögren’s Syndrome.

### 3.2 Construction and evaluation of the model

**Figure 3:**
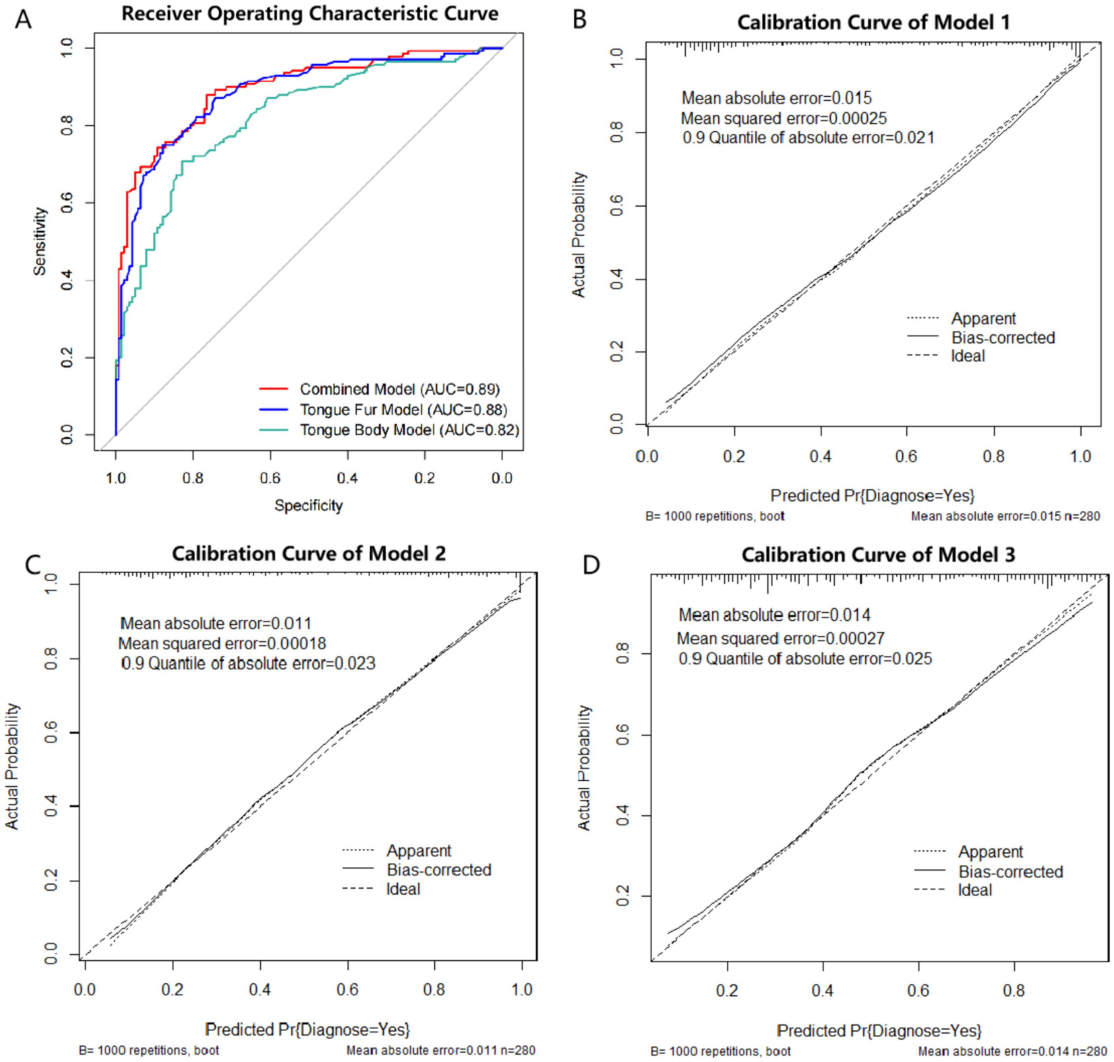
ROC curve and calibration curve of logistic regression model. **(A)** ROC curve of the three models. Red line: Model 1 (Combined model: tongue body and tongue fur), AUC = 0.89. Blue line: Model 2 (tongue fur model), AUC = 0.88. Green line: Model 3 (tongue body), AUC = 0.82. ROC: receiver operating characteristic; AUC: area under curve. **(B)** Calibration curve of Model 1 (Combined model: tongue body and tongue fur). **(C)**: Calibration curve of Model 2 (tongue fur model). **(D)**: Calibration curve of Model 3 (tongue body).

After variable selection, three models were constructed in this study. The tongue combined model is represented by Model 1: log(p/(1-p)) = 0.040 * age + 0.835 * sex + 0.561 * bluish-purple tongue + 1.219 * thin tongue + 1.089 * fissured tongue − 2.023 * thin fur + 2.346 * peeling fur − 2.498. The tongue fur model is represented by Model 2: log(p/(1–p)) = 0.053 * age + 0.795 * sex - 2.018 * thin fur + 2.926 * peeling fur − 2.502. The tongue body model is represented by Model 3: log(p/(1–p)) = 0.053 * age + 0.764 * sex + 0.583 * bluish-purple tongue + 1.101 * thin tongue + 1.644 * fissured tongue − 4.219. AIC is a widely used model selection criterion, where, in comparing multiple models, the one with the smallest AIC value is generally considered the best model. Among the three models, Model 1 has the smallest AIC value (Model 1: 244.86 < Model 2: 253.19 < Model 3: 303.13) (see Table 2). ROC curves for all three models are shown in Figure 3, with Model 1 exhibiting the largest area under curve (AUC) (Model 1: 0.89 > Model 2: 0.88 > Model 3: 0.82). The Brier score is a metric that evaluates the calibration accuracy of a classification model by measuring the difference between the predicted probabilities and the actual observed outcomes. In this context, Model 1 showed the largest AUC and the lowest Brier score (Model 1: 0.13 < Model 2: 0.14 < Model 3: 0.17), indicating superior discriminative ability and calibration accuracy compared with Models 2 and 3 (Figure 3A). Calibration curves, visualizing the calibration performance of classification models, were drawn using the bootstrap method on the training set, indicating good calibration performance for all three models (Figures 3B–D). In summary, model 1 was identified as the optimal model for diagnosing pSS based on tongue images in this study. The ROC curve of the training set revealed that the best cutoff value for diagnosing pSS with Model 1 was 0.347. The sensitivity, specificity, positive predictive value (PPV), negative predictive value (NPV), Youden’s Index and for diagnosing pSS using the optimal model in the training set were 87.86, 76.43, 78.85, 86.29%, and 0.64, respectively.

**Table 2:** Logistic regression model for Primary Sjögren’s Syndrome.

	Model 1		Model 2		Model 3	
	Beta	*P* value	Beta	*P* value	Beta	*P* value
Constant	−2.498	0.003	−2.502	0.002	−4.219	<0.001
Age	0.040	0.005	0.053	<0.001	0.053	<0.001
Sex at birth (female)	0.835	0.121	0.795	0.132	0.764	0.110
Bluish-purple tongue	0.561	0.099	/	/	0.583	0.048
Thin tongue	1.219	0.050	/	/	1.101	0.054
Fissured tongue	1.089	0.006	/	/	1.644	<0.001
Thin fur	−2.023	<0.001	−2.018	<0.001	/	/
Peeling fur	2.346	<0.001	2.926	<0.001	/	/
AIC	244.86	/	253.19	/	303.13	/

Model 1: Combined model (including tongue body and tongue fur); Model 2: tongue fur model; Model 3: tongue body model. AIC: Akaike information criterion.

### 3.3 Model validation

**Figure 4:**
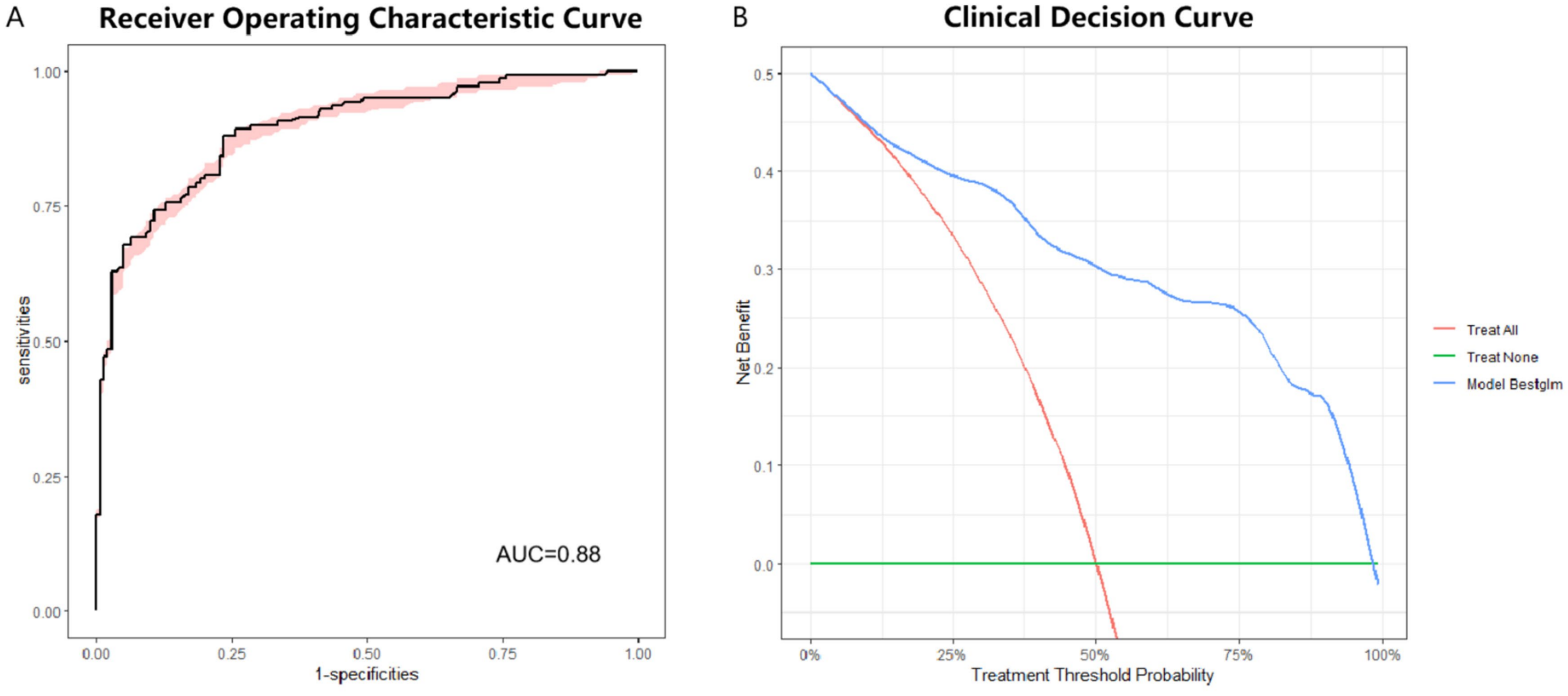
ROC curve and DCA curve for internal validation of optimal model. **(A)** ROC curve for internal validation of optimal model. AUC = 0.88. **(B)** DCA for internal validation of optimal model. ROC: receiver operating characteristic. DCA: decision curve analysis.

Internal validation of Model 1 performed by bootstrap. The results show good clinical prediction accuracy with an AUC of 0.88 (Figure 4A). DCA (Decision Curve Analysis) curves can assess the performance of predictive models in clinical decision making. The DCA for internal validation also showed good clinical applicability of Model 1 (Figure 4B).

### 3.4 Model testing

**Figure 5:**
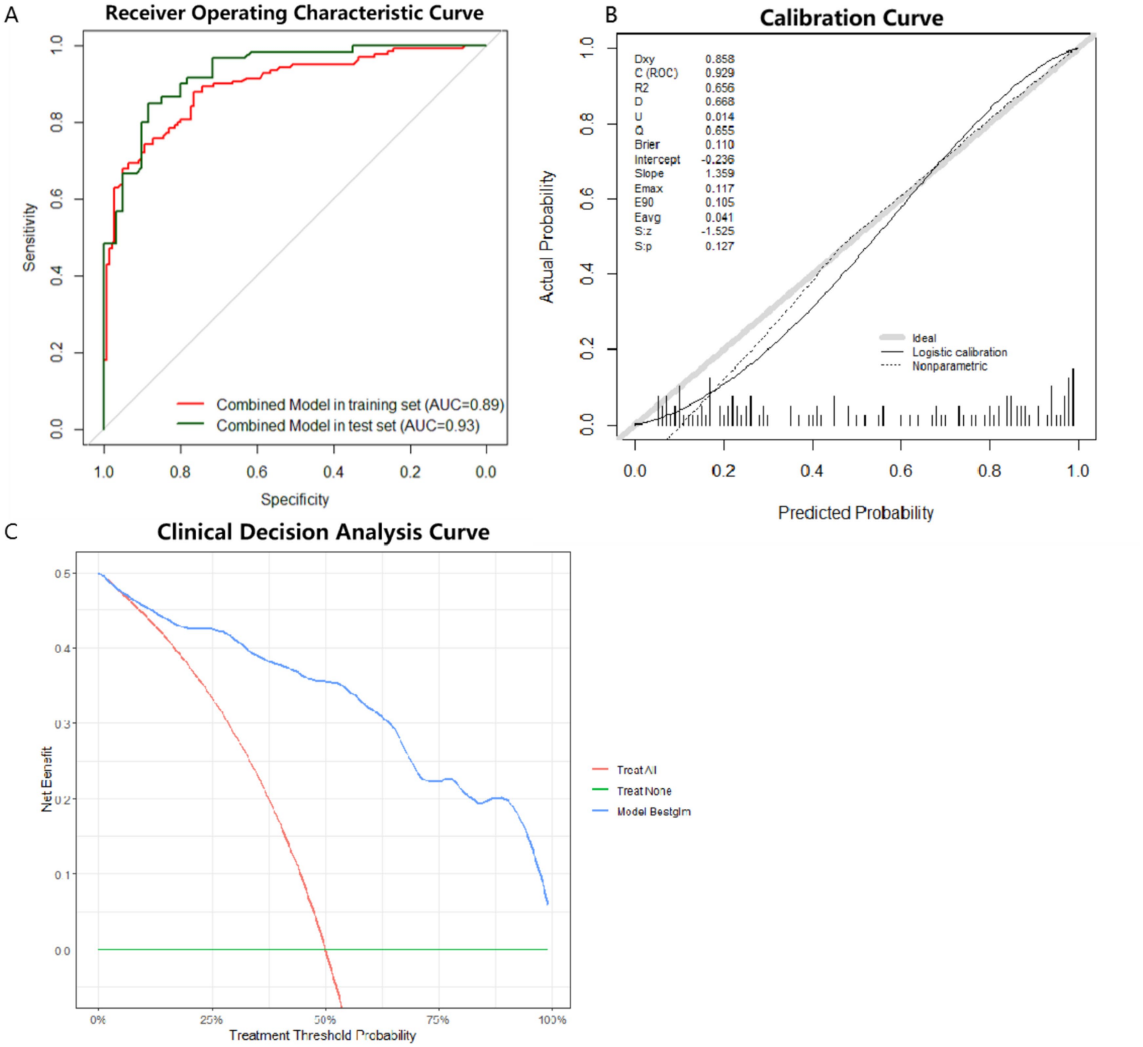
ROC curves, calibration curves and DCA curve for the optimal model in the test set. **(A)** ROC curve for external testing of optimal model. AUC = 0.93. **(B)** Calibration curve for external testing of optimal model. **(C)** DCA for external testing of optimal model. ROC: receiver operating characteristic. DCA: decision curve analysis.

Model 1 was subject to external testing using the test set. ROC curves, calibration curves and DCA were plotted for Model 1 (Figure 5). The results of AUC also showed that Model 1 exhibited high diagnostic accuracy on the test set (Figure 5A). When applying a cutoff value of 0.347 for diagnostic determination to the test set, the model showed sensitivity, specificity, PPV, and NPV for pSS of 93.33, 71.67, 76.71, and 91.48%, respectively. The Model 1 showed the best accuracy for the calibration curve (Figure 5B). DCA showed good clinical applicability of Model 1(Figure 5C).

### 3.5 Model presentation

**Figure 6:**
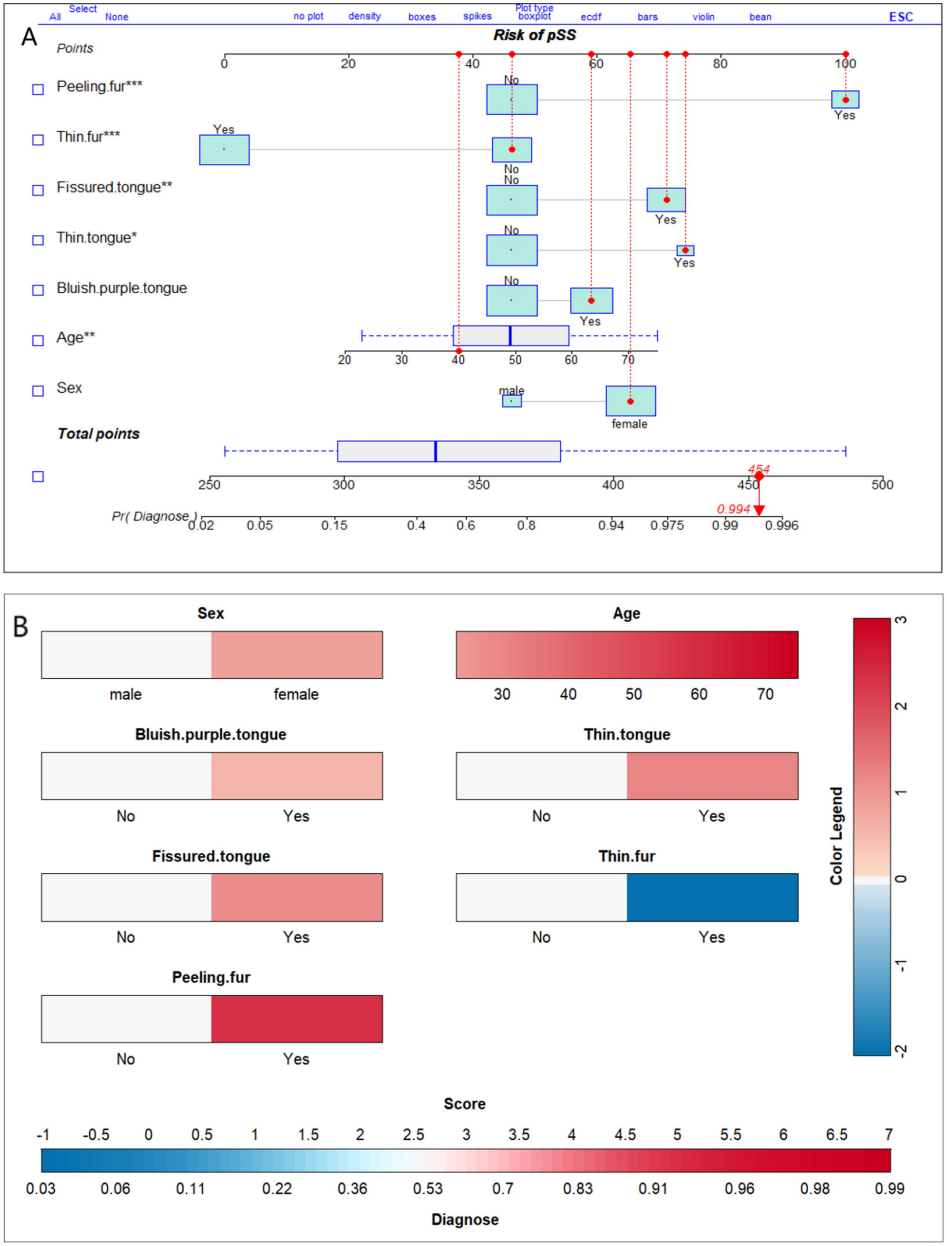
Nomograms of the optimal model for diagnosis of primary Sjögren’s syndrome based on tongue manifestations. **(A)** Depicts the probability of diagnosis of pSS in a 40-year-old female who displays peeling fur; a fissured, thin, and bluish-purple tongue; but not thin fur. **(B)** is similar to **(A)**, both of which can be utilized for diagnosing probabilities in a clinical setting. The different colors in **(B)** represent distinct scores and diagnostic probabilities. Initially, the color was selected based on the individual’s tongue manifestation, gender, and age. Then, we referred to the Color Legend to determine the corresponding scores. Next, all the scores were summed up and the corresponding diagnostic probability was located on the Score-Diagnose probability chart.

We present the optimal model using nomograms (Figure 6). Figure 6A shows an example of a 40-year-old female who has peeling tongue fur and a fissured, thin, and bluish-purple tongue, but not thin fur. The individual’s gender (female) corresponds to a score 65 points, with her age (40 years) corresponding to 38 points, the presence of peeling tongue fur to 100 points, fissured tongue to 71 points, thin tongue to 74 points, purplish tongue to 59 points, and absence of thin coating to 46 points. The total score for this individual is approximately 454 points, resulting in a pSS diagnosis probability of 0.994. Given the high diagnostic probability, we recommend that this individual undergo MSGB or serum auto-antibody testing for a definitive diagnosis. We also constructed a risk score prediction model represented by a colored nomogram (Figure 6B).

## 4 Discussion

Changes in tongue manifestations are commonly observed in patients with pSS (27). In this study, an optimal diagnostic model for pSS was constructed for the first time based on TCM tongue signs. The model underwent both internal and external validation, and a detailed diagnostic nomogram was drawn. The results showed that the diagnostic model for pSS based on sex, age, bluish-purple tongue, thin tongue, fissured tongue, thin fur, and peeling fur exhibited good differentiation and calibration, suggesting that the model has good diagnostic ability. Furthermore, the model has high sensitivity and specificity, with a cutoff value of 0.347, and the nomogram drawn based on the model can provide a clinical reference for noninvasive differentiation between normal individuals and those with pSS. This discovery provided readers with a new approach to the clinical diagnosis of pSS-an achievement that had not been accomplished before.

Tongue diagnosis is an efficient, non-invasive and convenient method that can support primary health care systems globally and can be performed anywhere as an aid to diagnosis (28). Traditional classification criteria for pSS involve numerous components, whereas the tongue model relies solely on visual examination of the patient’s exposed tongue. The DCA curve indicated that regardless of the probability of diagnosing pSS in patients, using the patient-friendly and economical pSS tongue model developed in this study to screen or diagnose suspected pSS patients could save a significant amount of healthcare costs while ensuring patient benefit. Inevitably, the subjective nature of physician interpretation of tongue images poses a challenge (23). In our study, the use of a standardized tongue imaging instrument, the establishment of a standard protocol for tongue images, and joint evaluations by two senior physicians ensured as much standardization as possible. For the first time, this study affirms the significant role of TCM tongue manifestations (tongue fur and tongue body) in screening or diagnosing pSS. The features we comprehensively considered include the tongue fur and the tongue body. The results showed that the combined tongue model was significantly better than the single tongue coating or tongue body model in terms of sensitivity and specificity, as well as better than previous study models that included only tongue color (18). Compared with prior studies that involved only female patients in the pSS tongue image diagnosis research (18), we included 400 research participants, encompassing both male and female subjects. This study further illustrated the applicability of tongue diagnosis in male pSS patients. To date, the model presented in this study exhibits the best sensitivity and specificity among the pSS tongue diagnosis model.

Tongue manifestations mainly include the tongue body and tongue fur. Tongue body manifestations comprise the appearance, color, and shape of the tongue, while the tongue fur refers to the thin layer of material covering the tongue, including the texture and color. In most healthy people, the tongue is light red in color with thin white fur. Owing to the involvement of salivary glands and parotid glands, the oral saliva content of pSS patients is significantly lower than that of normal people, and the composition of the former’s saliva is also altered, which directly affects the function of the tongue (29–31). In this study, it was found that people with bluish-purple tongue, thin tongue, fissured tongue, and peeling fur were more likely to be diagnosed with pSS, with the presence of fissured tongue and peeling fur being statistically significant. A fissured tongue is characterized by the presence of one or more shallow or deep grooves, furrows, or fissures on the surface of the tongue body. This tongue alteration was observed in tongue images of pSS patients of various races (32, 33). Furthermore, pSS patients with lymphoid malignancy exhibit a more fissured tongue (19). Peeling fur refers to the shedding of coating from the tongue surface, resulting in the loss of some or all of this covering from the tongue. Peeling of the tongue is usually one of the signs of glossitis. Oral candidiasis is a common oral infection in patients with pSS (34), and these patients may present with more severe tongue fur loss, or even present with a reddish tongue that is completely devoid of tongue fur (35). It is important to note that partial peeling of the tongue fur, known as geographic tongue, can also be possibly associated with psoriasis, and smoking (36, 37). In general, bluish-purple tongue includes not only a blue or purple tongue, but also localized dark spots or plaques on the tongue. In patients with pSS, there seem to be more localized dark spots or plaques than full tongue bruising or purpling, although this needs to be confirmed by more studies. Reduction of oral saliva and altered saliva composition in pSS patients lead to atrophy of the tongue body, which usually manifests as thin tongue. In this study, we found that thin tongue fur was negatively correlated with the diagnosis of pSS, and healthy individuals usually have thin tongue fur.

It is important to note that the appearance of tongue manifestations (tongue fur and tongue body) may be influenced by dietary factors, pharmacological agents, and lifestyle habits such as coffee consumption, bismuth-containing compounds, and smoking (38–40). To minimize confounding influences, participants were required to abstain from substances affecting the appearance of the tongue (including foods and medications) on scheduled imaging days, maintain complete fasting with avoidance of oral intake for 1 h preceding image capture, and disclose current pharmacotherapeutic regimens. Before using this model, clinicians should ask patients about their dietary habits and medication records to avoid observing incorrect tongue images.

The optimal diagnostic model showed that the likelihood of patients having the disease gradually increased with age, which is consistent with the findings of several previous studies (41). In the present study, the correlation coefficient for sex was higher, showing that the female population was more likely to be diagnosed with pSS. However, gender did not have a statistically significant correlation with pSS in this model, which may be attributed to the small sample size that was categorized as male in gender. Finally, this study presents the results in the form of nomograms, which provides a clearer and more convenient way of screening.

The present study also has some limitations. Only the Chinese population was included in this study. Although the results revealed by the tongue images were similar among various ethnic groups, this limits the generalization of the model to a certain extent. Furthermore, the present model only compares pSS patients with the healthy population, and future studies should evaluate the efficacy of the model in individuals suspected to have pSS. In addition, the study did not directly control for confounding factors affecting tongue manifestations. Diet and drugs can temporarily alter tongue manifestations, and the effect of these variables on tongue manifestations was tried in this study, but these variables were not assessed quantitatively in our current model.

## 5 Conclusion

This study established a diagnostic model for pSS based on specific tongue manifestations, age and sex, exhibiting high sensitivity and specificity with a cutoff value of 0.347. This model can be used as a reference for noninvasive clinical screening or diagnosing of pSS.

## Data Availability

The raw data supporting the conclusions of this article will be made available by the authors, without undue reservation.
